# Potential Effects of Sweet Potato (*Ipomoea batatas*) in Hyperglycemia and Dyslipidemia—A Systematic Review in Diabetic Retinopathy Context

**DOI:** 10.3390/ijms221910816

**Published:** 2021-10-06

**Authors:** Ruth Naomi, Hasnah Bahari, Muhammad Dain Yazid, Fezah Othman, Zainul Amiruddin Zakaria, Mohd Khairi Hussain

**Affiliations:** 1 Department of Human Anatomy, Faculty of Medicine and Health Sciences, Universiti Putra Malaysia, Serdang 43400, Malaysia; ruthmanuel2104@gmail.com (R.N.); haba@upm.edu.my (H.B.); 2 Centre for Tissue Engineering and Regenerative Medicine, Faculty of Medicine, Universiti Kebangsaan Malaysia, Cheras, Kuala Lumpur 56000, Malaysia; dain@ukm.edu.my; 3 Department of Biomedical Sciences, Faculty of Medicine and Health Sciences, Universiti Putra Malaysia, Serdang 43400, Malaysia; fezah@upm.edu.my; 4 Department of Biomedical Sciences, Faculty of Medicine and Health Sciences, Universiti Malaysia Sabah, Kota Kinabalu 88400, Malaysia; drzazakaria@gmail.com; 5 Halal Product Development Unit, Halal Product Research Institute, Universiti Putra Malaysia, Serdang 43400, Malaysia

**Keywords:** *Ipomoea batatas*, extraction, hyperglycemia, diabetic retinopathy, dyslipidemia, mechanism of action, signaling pathway

## Abstract

Hyperglycemia is a condition with high glucose levels that may result in dyslipidemia. In severe cases, this alteration may lead to diabetic retinopathy. Numerous drugs have been approved by officials to treat these conditions, but usage of any synthetic drugs in the long term will result in unavoidable side effects such as kidney failure. Therefore, more emphasis is being placed on natural ingredients due to their bioavailability and absence of side effects. In regards to this claim, promising results have been witnessed in the usage of *Ipomoea batatas* (*I. batatas*) in treating the hyperglycemic and dyslipidemic condition. Thus, the aim of this paper is to conduct an overview of the reported effects of *I. batatas* focusing on in vitro and in vivo trials in reducing high glucose levels and regulating the dyslipidemic condition. A comprehensive literature search was performed using Scopus, Web of Science, Springer Nature, and PubMed databases to identify the potential articles on particular topics. The search query was accomplished based on the Boolean operators involving keywords such as (1) Beneficial effect OR healing OR intervention AND (2) sweet potato OR *Ipomoea batatas* OR traditional herb AND (3) blood glucose OR LDL OR lipid OR cholesterol OR dyslipidemia. Only articles published from 2011 onwards were selected for further analysis. This review includes the (1) method of intervention and the outcome (2) signaling mechanism involved (3) underlying mechanism of action, and the possible side effects observed based on the phytoconstiuents isolated. The comprehensive literature search retrieved a total of 2491 articles using the appropriate keywords. However, on the basis of the inclusion and exclusion criteria, only 23 articles were chosen for further review. The results from these articles indicate that *I. batatas* has proven to be effective in treating the hyperglycemic condition and is able to regulate dyslipidemia. Therefore, this systematic review summarizes the signaling mechanism, mechanism of action, and phytoconstituents responsible for those activities of *I. batatas* in treating hyperglycemic based on the in vitro and in vivo study.

## 1. Introduction

The *Ipomoea batatas* (*I. batatas*) which is also commonly known as sweet potato is a plant that belongs to the Convolvulaceae family [1]. It is a perennial herbaceous vine that produces edible leaves and storage roots and can grow well on marginal lands. In this type of crop, asexual propagation is the most common form of dispersal, which results in a natural reduction of the genetic pool due to a lack of gene exchange [2]. *I. batatas* is a common staple root crop across the world as it produces the most edible energy per hectare and more than 6000 types of morphological variation of the leaf have been documented to date [3]. According to food and agriculture organizations of the United Nations in 2014, *I. batatas* is the sixth most consumed food crop worldwide, with a production of 178 million tons [4] and with China being the leading country in producing sweet potato [5]. The extraction of *I. batatas* contains a wide range of phenotypic variability [6] and bioactive compounds with acidic properties [7] and radical scavengers [8].

Additionally, in only the leaf, 130 known metabolites have been discovered through a simple extraction process [2]. Of those, phenolic compounds, such as mono caffeoylquinic acid, dicaffeoylquinic acid, and 3,4,5-tri caffeoylquinic acid, flavonoids including quercetin, myricetin, luteolin, and apigenin, and anthocyanins, are predominantly found in the leaf extracts [1]. In particular, dicaffeoylquinic acid and 3,4,5-tri caffeoylquinic acid possess anti-diabetic capacity and can inhibit lipid peroxidation [1]. Specifically, isolation of the caffeic acid derivatives, anthocyanosides, flavonoids, and arabinogalactan-protein from the *I. batatas* is said to be an effective anti-diabetic agent [9].

### 1.1. Hyperglycemia

Hyperglycemia is a condition in which the blood plasma contains an abnormal amount of glucose. It occurs when the body is unable to produce enough insulin or develops resistance to insulin. Hyperglycemia is when the blood glucose is greater than 125 mg/dL when fasting [10]. Hyperglycemia is the hallmark of diabetes mellitus, a chronic condition characterized by increased levels of glucose and an altered level of usual metabolic process in lipid and protein [11]. Many organs can be affected in the case of uncontrolled glucose levels. However, under hyperglycemic conditions, most cells can adjust to the rate of intracellular transport of glucose and protect the intracellular milieu from undesirable effects. However, certain cells, such as neural cells, endothelial cells, and β-cells, fail to stimulate glucose afflux regulation and equilibrate their intracellular glucose level to the extracellular concentrations, making them more vulnerable to the effects of hyperglycemia [12]. In hyperglycemic conditions, the production of nicotinamide adenine dinucleotide (NADH) production will be increased as a consequence of the increased level of glucose flux in the glycolytic pathway, causing a surge in the production of pyruvates and acetyl-CoA [13].

Since NADH is an electron carrier, an excessive level of NADPH will stress the mitochondrial electron transport chain which may not be inhibited by glucose-6-phosphate. As a result, the more glucose is consumed, the more glucose 6-phosphate (G6P) is produced, which is then broken down through glycolysis and the Krebs cycle, resulting in the uncontrolled formation of more NADH [14], eventually leading to the activation of NADPH oxidase. The enzymatic source of reactive oxygen species in the retina is NADPH oxidase, which is involved in the development of diabetic retinopathy. As a consequence, in a hyperglycemic state, microvascular complications of the retina can develop, which may induce oxidative damage in the retina, leading to the leakage of the tiny blood vessels, and act as signaling molecules to stimulate neovascularization, resulting in new fragile vessels [15].

### 1.2. Dyslipidemia

Dyslipidemia or lipid abnormalities are characterized by an increase in plasma cholesterol, triglycerides (TGs) in the range >200 mg/dL, or a reduction in high-density lipoproteins (HDL) cholesterol (<40 mg/dL) [16]. It is a complex disorder that involves central and specific organ mechanisms [17]. These include single or multiple gene mutations that result in either overproduction or defective clearance of triglycerides and low-density lipoproteins (LDL), or in the underproduction or excessive clearance of HDL. Similarly, other factors including a sedentary lifestyle along with excess consumption of calories, saturated fat, cholesterol, and trans fat diet may trigger this disorder [18]. Low-density lipoprotein cholesterol (LDL-C) components appear extremely small and compact, and more vulnerable to oxidation in the presence of hypertriglyceridemia (hyper-TG). Glycation of LDL-C is promoted by hyperglycemia, while the simultaneous glycation and oxidation of LDL-C will eventually raise the atherogenicity of LDL-C [19].

A recent cohort study shows that the prevalence rate of hypercholesterolemia in Malaysia in the years 2006 to 2012 (TC ≥ 240 mg/dL) was 44.9% while ethnic Malay tops the list with 51% [20]. Interestingly, a strong correlation was discovered between rising cholesterol levels in insulin-dependent diabetics and the severity of diabetic retinopathy. This means that people with high levels of total cholesterol or LDL-C in their serum are more likely to develop retinal hard exudate. This happens when the lipoproteins start to leak out from the retinal capillaries into the extracellular space of the retina, causing the formation of hard exudate in the retina [21].

## 2. Methods

### 2.1. Search Strategy

The search strategy was done based on the Preferred Reporting Items for Systematic Reviews and Meta-Analyses (PRISMA) guidelines described elsewhere [22]. A systematic review for the literature search was performed to identify relevant articles on the therapeutic potential of *I. batatas* leaves in hyperglycemia and dyslipidemia in regards to diabetic retinopathy. A literature search was performed comprehensively in a few selected databases such as Scopus, Web of Science, Springer Nature, and PubMed. The literature search was strictly selected for any publications related to the topic from 2011 onwards. The search query was done based on the Boolean operators, as described elsewhere [23]. The set of keywords are as follows: (1) Beneficial effect OR healing OR intervention AND (2) sweet potato OR *Ipomoea batatas* OR traditional herb AND (3) blood glucose OR LDL OR lipid OR cholesterol OR dyslipidemia.

### 2.2. Inclusion Criteria

Only research articles based on in vitro and in vivo studies that discussed effects, therapeutic potentials, signaling mechanisms, and mechanism of action were chosen to be further analyzed. The chosen articles had to be written in the English language with abstracts. The selected studies had to evaluate at least the following essential measures: (1) methods of intervention and outcome (2) signaling mechanisms involved (3) underlying mechanisms of action, and the possible side effects observed. Moreover, the reference was limited to within the last 10 years of publication, starting from January 2011 until June 2021.

### 2.3. Exclusion Criteria

Conference papers, thesis dissertations, review papers, manuscripts that were not written in English language, papers that did not have abstracts and articles that did not meet the criteria of the above-mentioned inclusion criteria were excluded. Studies focusing on *I. batatas* in clinical trials or other diseases, such as carcinoma, were also excluded from this review.

### 2.4. Data Extraction and Management

According to the inclusion criteria of this paper, all the published manuscripts were screened thoroughly. To ensure the guideline was followed, the titles and abstracts of each chosen articles were reviewed. Then, the full text was read to rule out articles that did not fulfill the inclusion criteria. Three independent reviewers (R.N., H.B., and M.D.Y.) did the initial screening of the titles and the abstracts of all identified records for potentially relevant studies. Any disagreement was discussed between the authors. The fourth reviewer (M.K.H.) was consulted to settle any form of disagreement that may arise. The obtained results include (1) the subject type, (2) dosage of treatment, (3) method of extraction, (4) follow up duration, (5) study outcome of the parameter assessed, and (6) the end result of the study. No conflict of interest was found among the reviewers during the data extraction process.

### 2.5. Strategy for Data Extraction

The findings of this literature review focus on in vivo trials of *I. batatas* in hyperglycemia and dyslipidemia in relation to the dosage of the treatment and the outcome observed. This is tabulated in Section 3. In Section 4, the analysis of the results is presented considering the mechanism of action and the signaling pathway involved in combating hyperglycemia and dyslipidemia with regards to diabetic retinopathy with some suggestions for future applications in standardized approaches that have been described extensively.

## 3. Results

### 3.1. Literature Search

**Figure 1:**
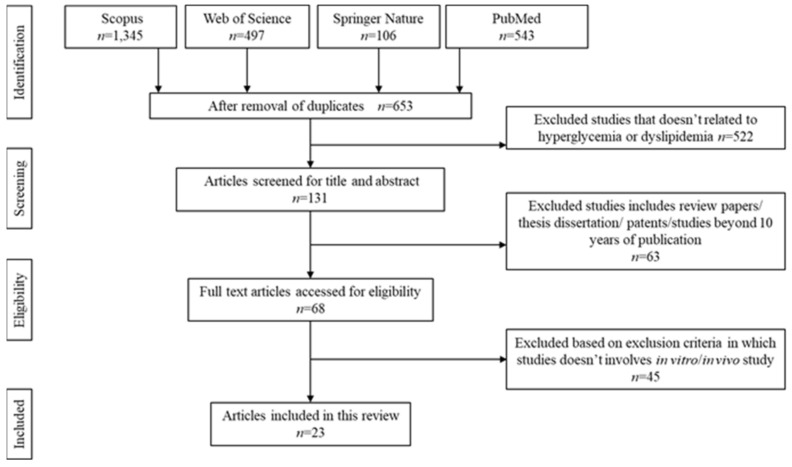
Identification and screening for literature search.

The literature search was able to identify 2491 articles that were potentially relevant to the title. Upon detection of duplication, 1838 articles were removed. Upon thoroughly screening the title and abstracts, 522 articles were removed. Due to unmatched content based on the inclusion criteria stated above, another additional 63 articles were excluded. Another 45 articles were rejected upon full-text screening either due to the constraint sample size or involvement of clinical trials. Deep screening allowed us to identify the final 23. The flow chart of the screening, identification, and the reasons for exclusion are summarized in Figure 1. Data extraction was done based on the finalized articles selection, as shown in Table 1 and Table 2.

### 3.2. The Diabetic Retinopathy as a Consequence of Hyperglycemia

Diabetic retinopathy arises as a complication of hyperglycemia and dyslipidemia. Particularly, the hyperglycemic conditions can induce microvascular damage through multiple pathways. This includes the hexosamine pathway, protein kinase C (PKC) pathway, polyol pathway, and the accumulation of advanced glycation end products (AGEs). However, the outcome of all these pathways manifests in a similar pathology which starts with alteration in the normal flow of blood in the retina [47].

A consequence of high blood glucose is nitric oxide, which is a vasodilator. As a result, the retinal blood vessels will start to dilate and the normal flow of blood to the retina is altered. Thus, metabolic autoregulation is initiated in the retina which may stimulate retinal metabolism [48]. Uncontrolled dilation of the blood vessel will eventually weaken the capillary wall [49]. In this case, the intraluminal pressure will become unbalanced, leading to the formation of microaneurysm [50], and thus the drastic apoptotic of pericytes [49]. Loss of pericytes triggers the formation of acellular capillaries in which the tubes are produced with the basement membrane, causing the capillary to occlude [51]. Over time, this vessel blockage will increase their permeability, which in turn will stimulate endothelial cell proliferation in the intravessel. As a corollary, neovascularization occurs, resulting in hemorrhages and vision loss [52].

On the contrary, the correlation between dyslipidemia and diabetic retinopathy is interrelated with AGE and the PKC pathway in the hyperglycemic condition. In the PKC pathway, glucose flux is raised via the glycolysis process. As a result, the key activator of PKC, de novo synthesis of diacylglycerol (DAG) will be stimulated. Consequently, the accumulation of long-chain fatty acids will be transformed into DAG as well, which will eventually upregulate PKC [53]. After, proteins in the extracellular matrix (ECM) will undergo differential synthesis causing the ECM to remodel. Along with these changes, there will be a sudden increase in the release of angiogenic factors and endothelial cells, leading to the dysfunction of leukocytes. Hence, the capillary is blocked and the normal blood flow to the retina is altered, resulting in vision loss [54].

**Figure 2:**
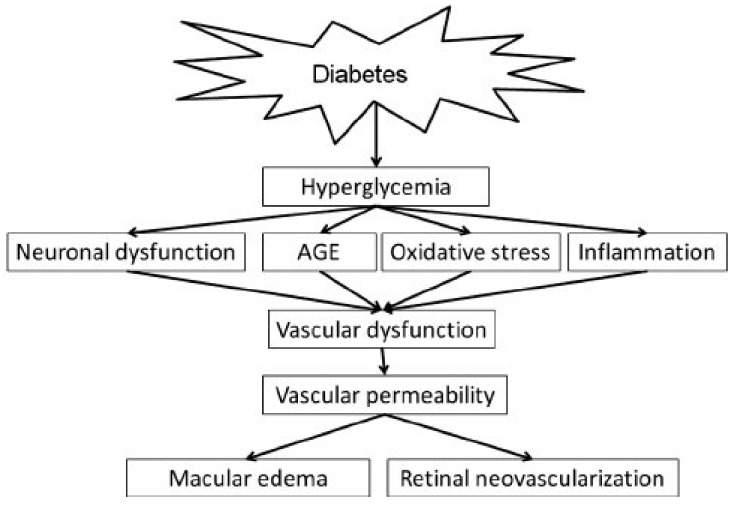
The pathophysiology of diabetic retinopathy as a consequence of high blood glucose level. Picture reproduced from Shin et al., (2014) [57]. Permission adapted from https://creativecommons.org/licenses/by-nc-sa/3.0/.

Similarly, AGE is closely associated with lipids and the development of vision loss. Non-enzymatic interactions between reducing sugars and lipoproteins produce AGE. In hyperglycemic condition, the level of AGE is increased drastically due to the availability of excessive glucose [55]. In a highly oxidative environment such as the retina, the accumulation of lipid and modification of protein will cause an accumulation of lipoxidation end products (ALEs), leading to the loss of pericytes, which in turn contributes to vascular complication, particularly in the retina [56]. Figure 2 summarizes the pathway of hyperglycemia leading to diabetic retinopathy.

### 3.3. Mechanism of Ipomoea batatas in Hyperglycemia and Dyslipidemia

The primary reason for *I. batatas’s* healing capability is mainly due to its bioactive compounds such as flavonoids and phenols. In this context, flavonoids are able to promote glucose absorption in peripheral tissue and enhance insulin secretion via the modulation of pleiotropic mechanisms [58]. The sequence of pleiotropic mechanisms includes stimulation of glucagon-like peptide-1 (GLP-1), which may enhance autonomic nerve activation and cause a rise in portal GLP-1. As a result, glucose synthesis via portal GLP-1 receptors will be hindered. At the same time, islet activities will be inhibited. Thus, the inactivation of locally produced intact GLP-1 in the islets is prevented. This may increase insulin secretion while suppressing glucagon secretion and possibly reducing islet inflammation simultaneously [59]. Certainly, being a natural antioxidant, flavone compounds in *I. batatas* extract are able to suppress reactive oxygen species (ROS) [60]. It has been hypothesized that the buildup of ROS due to persistent hyperglycemic condition is a major factor in the apoptosis of pancreatic β-cells in diabetes type 1 and insulin resistance in diabetes type 2 [61]. In addition, quercetin triggers the regeneration of β-cells in the pancreas, causing an increase in insulin secretion [62]. This is because quercetin has the ability to regulate Ca^2+^ fluxes. This in turn increases the intracellular concentration of Ca^2+^. As a result, the cellular pathway involving insulin secretion will be stimulated, reducing the possibility of insulin resistance, acting as an anti-diabetic agent [63].

Likewise, it is likely that the insoluble dietary fiber in *I. batatas* prevented lipid absorption in the small intestine [41]. By altering lipid absorption and transport, flavonoids may help to ameliorate dyslipidemia. At the gastrointestinal level, flavonoids enhance a drastic reduction of fat absorption. This is achieved via the regulation of different enzymes involved in lipid metabolism and the expression of transcription factors involved in triglyceride and cholesterol synthesis, such as sterol regulatory element-binding proteins (SREBP-1) and (SREBP-2). Through this mechanism, flavonoids could lower plasma triglycerides, total cholesterol, LDL cholesterol, and increase HDL cholesterol [64]. A similar mechanism is witnessed in the quercetin compound. In this case, by reducing pancreatic lipase activity, the quercetin reduces intestine dietary fat absorption. In addition, quercetin was found to inhibit cholesterol absorption through epithelial cholesterol transporters and reduce triglyceride absorption through epithelial fatty acid transporters such as fatty acid transport protein 4 (FATP4) [65]. Table 1 and Table 2 summarize the effect of *I. batatas* on hyperglycemic condition and dyslipidemia.

### 3.4. Signaling Mechanism of Ipomoea batatas in Reducing Hyperglycemic Condition

*Ipomoea batatas* is able to ameliorate hyperglycemic and regulate dyslipidemia via various signaling mechanisms. For example, it has been hypothesized that *I. batatas* has the capacity to lower tumor necrosis factor alpha (TNF-α) levels while also decreasing the expression of p38 mitogen-activated protein kinase (p38 MAPK) [24], a protein kinase implicated in β-cell death as well as influencing cellular responses to cytokines. Overexpression of TNF-α can activate the kinase by upregulating p38 phosphorylation, reducing the expression of Bcl-2 and Bax, an apoptosis regulator of β cells [66]. In this, inhibition of the p38 pathway hinders the downregulation of Bcl-2 and Bax which reduces blood glucose levels by enhancing the role of endogenous antiapoptotic Bcl proteins, such as Bcl-2 and Bcl-xL, thus suppressing the cell response to glucose [67].

**Figure 3:**
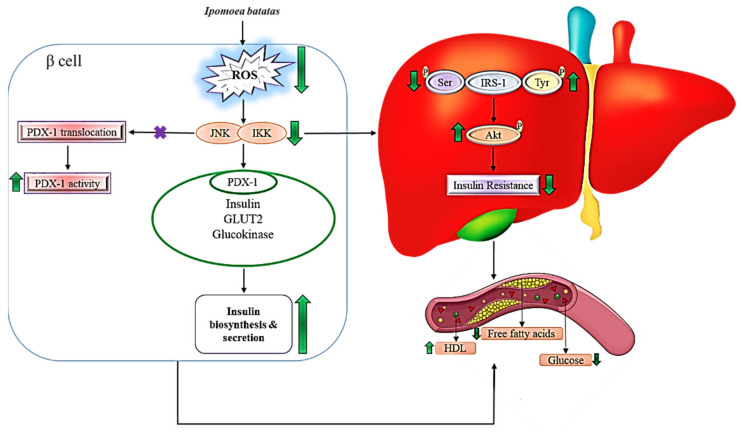
Mechanism of action of *Ipomoea batatas* in reducing hyperglycemia and regulating dyslipidemic condition.

Moreover, flavonoids in *I. batatas* improves glucose uptake by the cells via phosphoinositide 3-kinase (PI3K)/Akt and adenosine monophosphate-activated protein kinase (AMPK). The PI3K signaling pathway is a significant factor in the translocation of the glucose transporter (GLUT) protein from intracellular compartments to the plasma membrane, as it is a signal transduction system downstream of an insulin receptor (IR). The activation of various phosphorylation-dephosphorylation cascades occurs when insulin-IR binds within the cells. When this happens, the intracellular subunit of IRs autophosphorylates, the tyrosine kinase is activated, which catalyzes repeated phosphorylation of the IR substrate (IRS) proteins. In contrast, disruption in IRS protein phosphorylation or impaired PI3K recruitment from the cytosol, which results in PI3K inactivation, causes insulin resistance, followed by diabetes. In this situation, the flavonoids from *I. batatas* are able to activate Akt [36]. Activation of Akt further stimulates the translation of GLUT4, which is mainly expressed in the insulin-responsive tissues. As a result, insulin stimulates GLUT4 translocation from the intracellular locations to the cell surface, which enhances glucose absorption in cells, thereby reducing glucose levels in the plasma [68]. Figure 3 explains the signaling mechanism of *I. batatas* in reducing high glucose level and regulation of the dyslipidemic condition.

Similarly, a high level of glucose may cause the accumulation of ROS in β-cells. Accumulation of ROS may activate the c-Jun N-terminal kinase (JNK) pathway which may cause pancreatic and duodenal homeobox-1 (PDX-1) to translocate from the nucleus to the cytoplasm, resulting in a decrease in PDX-1 activity. This will eventually reduce the expression of the insulin gene. Thus, the normal insulin biosynthesis process is hindered [69]. In addition, the presence of quercetin in *I. batatas* ensures proper regulation of Ca^2+^ concentration in insulinoma cells. This usually correlates with TNF-α accumulation. This pathway may be activated by increasing the glucose concentrations, as in hyperglycemia. This is because a high level of TNF-α in insulinoma cells will raise the concentration of Ca^2+^cytosolic, which in turn stimulates calpain and calcineurin [70]. Activated calcineurin mediates dephosphorylation of the Bcl-2 associated with the agonist of cell death (BAD) protein. Such events will promote caspase activation, which could lead to pancreatic β-cell death via Ca^2+^ channel activation, thereby inhibiting the secretion of insulin [71]. On the contrary, calcineurin indirectly promotes insulin release by stimulating insulin gene expression. This action is mediated by the calcineurin-dependent dephosphorylation and activation of the transcription factor known as the nuclear factor of activated T cells (NFAT). However, calcineurin B1 deficiency may contribute to the development of diabetes mellitus as a result of insufficient insulin synthesis as age progresses. The results show that calcineurin can promote both anti- and pro-apoptotic processes in the same islet cell. Although calcineurin deficiency can lead to diabetes, research demonstrates that calcineurin inhibition is necessary to protect against cytokine-induced β-cell death [72].

### 3.5. Signaling Mechanism of Ipomoea batatas in Regulating Dyslipidemia

The presence of active compounds such as polyphenols in *I. batatas* is known to be an essential element in regulating dyslipidemia. It has been hypothesized that polyphenols could suppress fat accumulation via the downregulation lipogenic pathway particularly via the downregulation of SREBP-1c and its downstream molecules, specifically lipogenic genes such as acetyl-CoA carboxylase (ACC) and stearoyl-CoA desaturase (SCD) [73]. When the lipogenic genes, ACC and SCD, are being suppressed, the concentration of malonyl-CoA will be reduced. As a result, carnitine palmitoyl transferase (CPT)-1 will be activated, enhancing the β-oxidation of fatty acids [74]. Thus, the transport of non-esterified fatty acids to the liver will be inhibited, leading to the reduction in the triglyceride synthesis [75]. Additionally, deleting or suppressing ACC further decreases fat mass and increases insulin sensitivity via the lowering of malonyl-CoA levels [76]. Meanwhile, SCD reduces fat accumulation by catalyzing the biosynthesis of monounsaturated fatty acids from saturated fatty acids [77].

## 4. Discussion

The findings obtained in this review are shown in Table 1, highlighting the beneficial effect of *I. batatas* as an anti-hyperglycemia agent [24,25,26,27,28,29,30,31,32,33,34,35,36,37,38,66,67,68,69,70,71,72,73,74,75,76,77] and also a good regulator in dyslipidemic conditions [39,40,42,43,44,45,46]. A positive outcome in reducing plasma glucose level is witnessed as early as six days after treating diabetic rats with *I. batatas* extract [34], while 28 days of continuous treatment with *I. batatas* extract is considered an ideal duration for the extract to exhibit a maximum level of effect. In regards to this statement, three experimental studies conducted for up to 28 days observed regeneration of β-cell mass [24]. A minimum amount of 100 mg seems to be effective in revealing *I. batatas* healing ability in hyperglycemic conditions [25]. Meanwhile, the findings obtained by all the researchers are almost identical to one another. In this case, all the studies reviewed show that *I. batatas* extract, regardless of the method of extraction, are able to reduce plasma glucose level, glycosylated protein, and increased level of insulin [24,25,26,27,28,29,30,31,32,33,34,35,36,37,38,66,67,68,69,70,71,72,73,74,75,76,77].

A study done by Niwa et al. in 2011 shows that a decreased level of the 8-OHdG marker in serum decreased formation of nitrotyrosine in the aorta [24]. 8-OHdGs is an important marker indicating severe-form diabetes, which is produced due to DNA oxidation in cells. This may lead to other secondary complications such as micro or macrovascular complications, most likely diabetic retinopathy [78]. Reduction of 8-OHdG level indicates *I. batatas* has the ability to hinder deoxyribonucleic acid (DNA) oxidation in β-cells. On the other hand, nitrotyrosine is an indicator for the formation of peroxynitrite which is considered a potent nitrating and oxidant agent. Therefore, a high level of nitrotyrosine may deplete the antioxidant defense and hinder the normal role of certain enzymes, which may trigger direct cytotoxic effects to endothelial cells, leading to apoptosis of fibroblasts and myocytes in the heart [79]. The outcome observed by Niwa et al., 2011 [24] show the protective ability of *I. batatas* anti-apoptotic agent. Similarly, Pal et al., 2015 observed an inhibitory effect on α-glucosidase in rats fed with ethanol extracted *I. batatas* [77]. Generally, α-glucosidase is an exotype carbohydrase and a membrane-bound enzyme found in the epithelium of the small intestine that catalyzes the hydrolytic breakdown of oligosaccharides into absorbable monosaccharides to aid glucose absorption by the small intestine. In other words, α-glucosidase catalyzes the release of glucose from the substrate’s non-reducing end. Hence, inhibition of α-glucosidase hinders the rise in glucose levels in plasma [80].

Nonetheless, *I. batatas* extract has proven to be a potent antioxidant. This claim was further proven when Lin et al., 2017 observed a high iron-chelating capability [26]. It is obvious that ROS accumulation mediates apoptosis of pancreatic islet cells, leading to a low level of insulin secretion, as β-cells are very susceptible to oxidative damage [81]. Systemic iron overload may dysregulate glucose metabolism [82] and generate an excessive amount of ROS via Fenton reaction [83]. Meantime, Tahir et al., 2018 witnessed a reduction in Ca^2+^ in various organs upon force-feeding streptozotocin-induced rats [27]. This shows that *I. batatas* has the ability to inhibit insulin resistivity, poor insulin sensitivity, and impaired glucose tolerance which is a complication of a high level of Ca^2+^ in serum [84]. Moreover, Akhtar et al., 2018 [28] and Kamal et al., 2018 [29] noticed a drastic reduction in SGPT and SGOT levels in the laboratory assessment. Both SGPT and SGOT are an indicator of tissue strain in the liver due to a high sugar level. A similar result was identified by Refaat et al., 2020 [35], Shih et al., 2020 [36], and Jiang et al., 2020 [37] who observed a reduction in ALT, AST, and ALP. The outcome shows the capability of *I. batatas* extract to exhibit a protective effect against the liver from ROS due to a high level of glucose.

Nevertheless, investigations done by Rafiu et al., 2018 [32], Almoraie 2019 [33], and Jiang et al., 2020 [37] showed that *I. batatas* could increase hepatic enzyme activity, specifically SOD, catalase, and GPx, and decrease the level of MDA in hepatocytes. With oxidative stress implicated in the pathogenesis of diabetic patients, reduction in the SOD, catalase, and GPx are a common pathology. Interestingly, all the findings in this review support the statement that these positive effects of *I. batatas* that are exhibited through several signaling mechanisms. For instance, Niwa et al., 2011 [24] conclude that *I. batatas* exhibits anti-hyperglycemic effects through the p38 MAP kinase signaling pathway while Luo et al., 2021 [37] state that the effects are manifested via PI3K and the glycogen synthase kinase-3β signaling pathway. However, in both pathways debated in their study, the pathway leads to the suppression of cell response to glucose, thereby reducing the glucose level in the plasma.

Conversely, all laboratory evaluations, as shown in Table 2, demonstrate almost similar outcomes on the role of *I. batatas* in regulating dyslipidemic conditions. One study chosen to be reviewed in this article shows that 0.3 mL of aqueous extraction is enough to disclose the underlying mechanism of *I. batatas* in regulating such conditions [39,40,42,43,44,45,46]. They notice that there is a decreased level of triglyceride, total cholesterol, and glucose in plasma while the level of HDL starts to rise over time. Although there is not much evidence on the underlying signaling mechanism involved, a recent study speculated that this could be due to the presence of *I. batatas* bioactive compounds such as flavonoids, quercetin, and polyphenols that are able to suppress fat accumulation via the downregulation of lipogenic pathway [73].

## 5. Safety Concern and Dosage Recommendation

*Ipomoea batatas* has been classified as a medicinal food. However, safety concerns in consuming *I. batatas* have still not been considered in certain conditions. For instance, *I. batatas* contains oxalic acid, an organic compound which may form oxalates stone in the urinary tract in dehydration condition [85]. In in vivo trials, no toxicity was encountered up to 5000 mg/kg of *I. batatas* extract ingestion. However, long-term administration of *I. batatas* extract at doses greater than 1000 mg/kg has been shown to have deleterious effects on the liver and kidney [86]. In contrast, clinical trials showed no serious adverse effects. However, *I. batatas* is not recommended for those with a hypersensitivity reaction, as those patients have a high possibility to develop generalized urticaria, hypotension, edema, dizziness, vomiting, sensation of tickling in the throat, and loss of consciousness upon consuming *I. batatas* [87].

## 6. Conclusions and Future Perspective

In conclusion, *I. Batatas* are a very versatile vegetable that can be used as a medication substitute. It is effective in treating hyperglycemia, and its activity is found to be higher than that of diabinese, a commonly used diabetes drug. Just within 8 weeks of *I. batatas* consumption, pancreatic cell function is readily increased, lipid levels reduced, insulin resistance starts to diminish, and reduced glycemic index is witnessed. The results prove that *I. batatas* could potentially be used to treat the hyperglycemic and dyslipidemic conditions. However, clinical trials are still lacking in evidence since not many clinical trials have been done on this extract. Research reveals no clinical data regarding the use of *I. batatas* in treating dyslipidemia, although a Cochrane review indicates that 4 g/day is safe for up to 5 months for hyperglycemic treatment. Yet, the long-term effect has not been studied yet. Therefore, it is suggested to further explore the potential of *I. batatas* in human trials for its potential role in bioprospecting and drug discovery in treating hyperglycemic and dyslipidemic conditions in future.

## Figures and Tables

**Table 1:** Effect of *I. batatas* on hyperglycemic condition.

Author	Type of Subject	Dosage of Treatment	Extraction Method	Follow Up Duration	Findings	Conclusion
Niwa et al., 2011 [24]	Male Wistar rats injected with Streptozotocin	5 g/kg/day for 8 weeks	Not stated	1st, 3rd, 5th, and 7th week	Increased of body weight. Decreased level of blood glucose. Decreased level of glycosylated hemoglobin subunit alpha 1 (HbA1c) in plasma. Decreased level of ketones in urine. Increased level of insulin in serum. Decreased level of superoxide production by leukocytes. Decreased level of 8-hydroxy-2′ -deoxyguanosine (8-OHdG)s marker in serum. Decreased formation of nitrotyrosine in aorta. Decreased level of TNF-α, and albumin in urine. Decreased level of p38 MAP kinase and phosphorylation in aorta. Increased level of pancreatic β-cell mass.	*I. batatas* exhibits anti-hyperglycemic effects through the p38 MAP kinase signaling pathway.
Pal et al., 2015 [25]	Male albino Sprague Dawley rats injected with Streptozotocin	100 mg/kg/day for 30 days	Ethanol and aqueous	7th, 14th, 21st, and 28th day	Improvement in fasting blood glucose and glucose tolerance (OGTT). Reduced blood glucose level. Decreased level of glycosylated HbA1c in plasma. Increased level of insulin in serum. Decreased level of cholesterol and triglycerides in serum. Decreased level of aspartate transaminase (AST), alanine Aminotransferase (ALT), urea, and creatinine in serum. Inhibitory effect is seen on α-glucosidase.	Aqueous extraction of *I. batatas* is an effective anti- hyperglycemic agent.
Lin et al., 2017 [26]	CN1927, CYY98 of sweet potato leaf extract (SPLE), and 40 °C oven-dried TN64	Not relevant	Ethanol	-	High diphenyl-β-picrylhydrazyl (DPPH) and 2,2 -azino-bis(3-ethylbenzothiazoline-6-sulfonic acid) (ABTS) radical-scavenging activity. High level of iron chelating capacity. Increased expression of insulin in hepatocytes. Increased level of Insulin receptor substrate in hepatocyte cells. Increased expressions of glucose transporter-2. Increased TNF-α.	SPLE improves TNF-α -induced insulin resistance by activating insulin signaling.
Tahir et al., 2018 [27]	Male Wistar rats induced with Alloxan monohydrate	4 g/kg/day for 15 days	Methanol and aqueous	3rd, 6th, 9th, 12th, and 15th day	Decreased level of calcium and magnesium in liver, kidney, heart, brain, lungs, forelimb, and hind limb.	*I. batatas* decreases diabetic complications by decreasing calcium and magnesium level in various organs.
Akhtar et al., 2018 [28]	Male Wistar rats induced with Alloxan monohydrate	4 g/kg/day for 14 days	Methanol	3rd, 6th, 9th, 12th, and 15th day	Decreased level of blood glucose. Decreased level of glycosylated protein. Decreased level of total cholesterol, triglycerides, and LDL-cholesterol. Increased level of HDL-cholesterol. Increased level of albumin and globulin concentration in liver. Decreased level of serum glutamic pyruvic transaminase (SGPT) and serum glutamic oxaloacetic transaminase (SGOT) in hepatocytes.	Methanol extraction of *I. batatas* is an effective anti-hyperglycemic agent.
Kamal et al., 2018 [29]	Male Wistar rats induced with Alloxan monohydrate	4 g/kg/day for 14 days	Aqueous	3rd, 6th, 9th, 12th, and 15th day	Decreased level of blood glucose. Decreased level of glycosylated protein. Decreased level of total cholesterol, triglycerides, and LDL-cholesterol. Increased level of HDL-cholesterol. Increased level of albumin and globulin concentration in liver. Decreased level of SGPT and SGOT in hepatocytes.	Aqueous extraction of *I. batatas* is an effective anti-hyperglycemic agent in older aged subjects.
Omodamiro et al., 2018 [30]	Adult female albino rats	1000 mg/kg/day or 750 mg/kg/day or 500 mg/kg/day or 250 mg/kg/day for 7 days	Methanol	Every 2 days	Decreased level of blood glucose. Decreased level of total cholesterol and triacylglycerol concentration in serum. Increased level of HDL concentration in serum.	Methanol extraction of 500 mg/kg is an optimum dose for anti-hyperglycemic.
Rafiu et al., 2018 [31]	Adult male Wister strains albino rats induced with Streptozotocin	400 mg/kg/day for 28 days.	Aqueous	Every day	Decreased level of blood glucose. Decreased level of urea, creatinine, and uric acid. Decreased level of packed cell volume (PCV), hemoglobin (Hb), and red blood cell (RBC) in serum. Increased level of neutrophils. Decreased level of platelet, white blood cell (WBC), lymphocytes, and eosinophils. Increased level of basophils and monocytes.	Aqueous extraction of 400 mg/kg is an optimum dose for anti-hyperglycemic.
Rafiu et al., 2018 [32]	Adult male Wister strain albino rats induced with Streptozotocin	400 mg/kg/day for 28 days.	Aqueous	Every 2 days	Decreased level of blood glucose. Increased level of glycosylated protein in serum. Decreased level of triglyceride in serum. Decreased level of total cholesterol in serum. Increased level of HDL. Increased level of sodium, chloride, bicarbonate, phosphates and calcium in serum. Decreased level of lipid peroxidase and Malondialdehyde (MDA) in serum. Increased level of catalase and superoxide dismutase (SOD) in serum. Decreased level of glutathione and glutathione-s-transferase in serum.	*I. batatas is* able to reverse hyperglycemic condition.
Almoraie, 2019 [33]	Adult male Wister albino rats induced with Streptozotocin	200 mg/kg/day for 4 weeks	Aqueous	Not stated	Decreased level of blood glucose. Decreased level of pancreatic MDA. Increased level of glutathione peroxidase (GPx) and SOD in serum. Decreased level of interleukin-1β (IL-1β) and TNF-α. Diminished of cell necrosis in islets of Langerhan’s. Reverse action of hypertrophy in Langerhans islets.	*I. batatas is* able to reverse hyperglycemic condition, hyperinsulinemia, oxidative stress, inflammatory and histopathological changes in pancreas.
Novrial et al., 2020 [34]	Male Sprague Dawley rats induced with Streptozotocin	0.25 g/kg/day or 0.8 g/kg/day or 2.5 g/kg/day for 14 days	Ethanol	6th and 14th day	Reduced level of fasting blood glucose. Decreased level of blood glucose. Low grade of insulitis is seen in islets of Langerhans. Increased expression of β-cell insulin.	*I. batatas is an* effective hyperglycemic agent.
Refaat et al., 2020 [35]	Adult male albino Sprague Dawley rats induced with Alloxan monohydrate	2.5% or 5% of diet for 28 days	Powdered form (freeze dried)	7th, 14th, 21st, and 28th day	Decreased level of blood glucose. Decreased level of alanine amino transferase (ALT), aspartate amino transferase (AST), and alkaline phosphatase (ALP) in serum. Decreased level of creatinine, uric acid, and urea in serum. Decreased level of total cholesterol, triglycerides, LDL-c, VLDL-c, HDL-c in serum.	5% of grinded *I. batatas* leaves in diet is an effective anti-hyperglycemic agent.
Shih et al., 2020 [36]	Male mice induced with Streptozotocin	0.5% or 5% of diet for 8 weeks	Powdered form (freeze dried)	Once a week	Decreased level of plasma glucose. Increased level of insulin. Decreased level of ALT, triglyceride, and TNF-α. Significant restoration of the Langerhans’s areas. Increased expression of insulin-signaling pathway related proteins, phosphorylated insulin receptor, protein kinase B, and membrane glucose transporter 4.	*I. batatas* exhibits anti- hyperglycemic effects by stimulating the regeneration of pancreatic islet and insulin resistance.
Jiang et al., 2020 [37]	Male mice induced with Streptozotocin	500 mg/kg/day for 8 weeks	Ethanol	Once a week	Increased level of glucose tolerance and lipid metabolism. Decreased level of fasting blood glucose. Decreased level of ALT and AST. Decreased level of lipid peroxidase and MDA in serum. Increased level SOD in serum. Increased level of insulin in serum. Decreased level of glutathione and glutathione-s-transferase in serum. Decreased level of oxidative stress. Increased level of glucose transporter type 2, glucokinase, and insulin receptor α. Up regulation of phosphofructokinase and pyruvate kinase. Down regulation of glucose-6-phosphatase (G6PD) and phosphoenolpyruvate carboxykinase.	Sweet potato extract stimulates glycolysis and reduces gluconeogenesis.
Luo et al., 2021 [38]	Mice	Not specified	Not specified	Not specified	Increased level of oral glucose tolerance. Decreased level of fasting blood glucose. Increased level of fasting serum insulin. Decreased level of insulin resistance. Increased level of hepatic glycogen. Decreased level of total cholesterol, triglycerides, and LDL. Increased level of HDL. Inhibition of β-cell apoptosis. Up regulation of PI3K and glycogen synthase kinase-3β signaling pathway.	*I. batatas* exhibits anti-hyperglycemic effects through PI3K and glycogen synthase kinase-3β signaling pathway.

**Table 2:** Effect of *I. batatas* on dyslipidemia.

Author	Type of Subject	Dosage of Treatment	Extraction Method	Follow up Duration	Findings	Conclusion
Park et al., 2012 [39]	Male Sprague Dawley rats fed with high-fat diet (HFD)	5% of total diet for 4 weeks	Fleshed	4th week	Increased level of total cholesterol and triglyceride content in fecal. Decreased level of total cholesterol and triglyceride content in serum. Decreased level of LDL-cholesterol level.	Sweet potato extraction is an effective agent to enhance fecal lipid excretion.
Jawi et al., 2015 [40]	Local Balinese male rabbits fed with high cholesterol diet	4 mL/kg/day for 60 days	Aqueous	60th day	Decreased level of total cholesterol in serum. Decreased level of MDA. Decreased level of interleukin-1 (IL-1) in blood.	Sweet potato extract is able to reduce cholesterol content.
Kurata et al., 2017 [41]	Male Sprague Dawley rats fed with HFD	0%, 1%, 3%, and 5% of total diet for 35 days	freeze-dried powder	35th day	Decreased in body weight and food intake. Decreased in tissue weight of kidney, liver, and adipose tissue. Decreased level of triglyceride, total cholesterol, and glucose in plasma. Increased level of HDL in plasma. Increased level of antioxidant activity in blood.	*I. batatas* is able to stimulate lipid metabolism.
Nasoetion et al., 2019 [42]	Broiler chicks fed with crude protein diet	25 mL/kg/day and 50 mL/kg/day for 35 days	Aqueous	Weekly for 35 days	Increased growth rate, feed intake and feed efficiency. Decreased level of LDL, total cholesterol, and fat in blood and liver. Increased level of HDL in blood and liver. Decreased level of hepatic lipid metabolism.	Sweet potato extract is able to reduce fat deposition.
Nur et al., 2019 [43]	Zebrafish (Danio rerio) fed with HFD	80 ppm, 120 ppm, and 160 ppm for 40 days	Ethanol	Weekly for 40 days	Increased growth rate, feed intake and feed efficiency. Decreased level of glucose in blood. Decreased level of total cholesterol in plasma. Decreased level of Peroxisome proliferator- activated receptor gamma (PPAR-γ).	Sweet potato extract is able to reduce fat deposition.
Heriwijaya et al., 2020 [44]	White male Wister rats fed with high cholesterol diet	3 cc, 6 cc, and 9cc for 12 weeks	Aqueous	12th week	Decreased level of LDL, triglyceride, and total cholesterol. Increased level of HDL in blood.	Purple sweet potato leaf extract is able to reverse hyperlipidemia condition
Khairani et al., 2020 [45]	Male Mus Musculus mice fed with HFD	0.3 mL/day, 0.5 mL/day, and 1.0 mL/day for 9 weeks	Aqueous	9th week	Decreased level of cholesterol, triglycerides, and LDL in blood. Reduced weight of visceral fats and liver. Inhibition in the elevation of overall body weight. Increased in the lactic acid bacteria colony in gut.	0.3 mL/20 g/bw is the optimum dosage of the extract to exhibit maximum effect.
Ntchapda et al., 2021 [46]	Male Wistar rats fed with HFD	400 mg/kg, 500 mg/kg, and 600 mg/kg for 4 weeks	Aqueous	4th week	Decreased in body weight. Increased level of feces excretion. Decreased level of cholesterol, triglycerides, VLDL-c and LDL in blood. Increased level of HDL in blood. Decreased level of AST, ALT, glucose, and creatinine in serum.	*I. batatas* is able to reverse hyperlipidemia condition.

## Data Availability

Not applicable.
